# Split otoferlins reunited

**DOI:** 10.15252/emmm.201809995

**Published:** 2018-12-20

**Authors:** Jeffrey R Holt, Gwenaelle SG Geleoc

**Affiliations:** ^1^ Department of Otolaryngology Boston Children's Hospital and Harvard Medical School Boston MA USA

**Keywords:** Genetics, Gene Therapy & Genetic Disease, Neuroscience

## Abstract

Gene therapy for genetic hearing loss is a nascent field with just a handful of studies published to date that demonstrate proof‐of‐concept recovery of auditory function (reviewed in Ahmed *et al*, 2017; Lustig & Akil, 2018). One challenge that faces the inner ear field, as well as the broader gene therapy field, is the need to deliver large gene sequences despite the limited genetic capacity (~4.5 kB) of delivery vehicles such as adeno‐associated viral vectors (AAV). In this issue, Al‐Moyed *et al* have overcome this conundrum by using two AAV vectors to deliver the coding sequence for otoferlin, which is ~6 kB. With dual‐AAV delivery of split otoferlin and a trans‐splicing approach, they demonstrate recombination of full‐length otoferlin in sensory hair cells of the inner ear, enabling partial restoration of auditory function in deaf, otoferlin‐null mice.

The *OTOF* gene encodes otoferlin, a large calcium‐sensor protein required for synaptic transmission between mechanosensory hair cells in the inner ear and spiral ganglion neurons (SGNs), which carry sound information to the brain via the eight cranial nerves. Pathogenic mutations in *OTOF*/*Otof* cause profound deafness in humans (DFNB9) and mice (Yasunaga *et al*, 1999; Roux *et al*, 2006). Because otoferlin expression in the cochlea is restricted to a subset of cells known as inner hair cells (IHCs), therapeutic strategies for overcoming the consequences of *OTOF* mutations need only target a quarter of cochlear hair cells, ~4,000 in humans, ~1,000 in mice. Luckily, in this case, AAV vectors provide a strong advantage, since a number of different AAV serotypes have been identified that efficiently target cochlear inner hair cells (IHCs; Ahmed *et al*, 2017). Another important advantage for development of *OTOF* gene therapy is the fact that otoferlin is not required for development or survival of inner hair cells. Thus, although many DFNB9 patients are born with congenital deafness, their post‐mitotic sensory hair cells survive, suggesting there may be a postnatal window for clinical intervention.

Because of these advantages, Al‐Moyed *et al* (2019) were motivated to overcome the disadvantage of AAV vector capacity being too small to accommodate the otoferlin coding sequence. While other gene therapy vectors are available, most do not provide the requisite cell specificity, or their use has raised concerns due to integration into the host genome (i.e. retrovirus, lentivirus) or strong immune responses (i.e. adenovirus). Thus, the investigators opted to focus on AAV gene replacement strategies using full‐length *Otof* split in two (Fig 1). They generated dual‐AAV vectors with the two halves of the split *Otof* gene carrying complementary coding sequences. The goal was to reconstitute full‐length *Otof* in the doubly transduced target cells, the IHCs. Dual‐AAV trans‐splicing (TS) uses splice donors and acceptors placed on the 3′‐end and 5′‐end of the split *Otof* coding sequence, respectively. Upon co‐transduction of the same cell, the two halves undergo head‐to‐tail concatemerization or trans‐splicing to produce full‐length mature mRNA and hence full‐length protein. An alternate strategy, the dual‐AAV “hybrid” (Hyb), combines splice donors and splice acceptors within a highly recombinogenic region of an exogenous gene.

**Figure 1:**
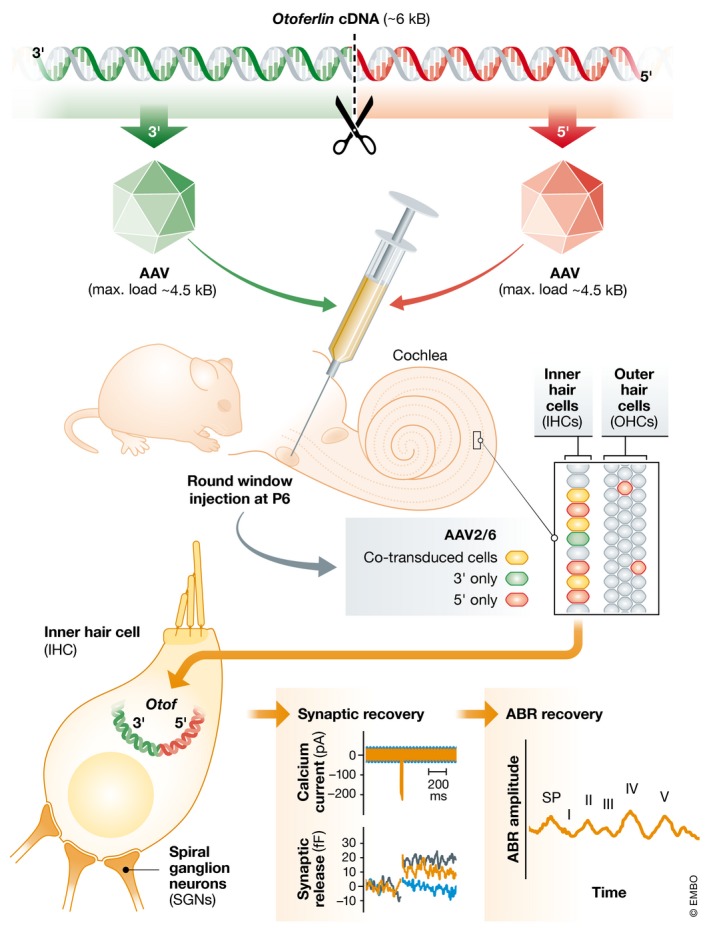
Dual adeno‐associated viral vectors (AAVs) for gene replacement in the inner ear Split AAVs, each containing a fragment of the large otoferlin coding sequence are injected into the inner ears of otoferlin knockout mice. Upon co‐transduction in single cells, the split sequences reassemble full‐length otoferlin, which in sensory hair cells leads to recovery of protein expression, partial recovery of synaptic function and auditory brainstem responses (ABR). Bottom panels illustrate synaptic and ABR recovery after dual‐AAV treatment (orange) relative to Otof^−/−^ mice (blue) and wild‐type mice (black).

Initial attempts for dual vector delivery in the retina targeted the large Usher syndrome deaf‐blindness gene, USH1B (encoding for myosin‐7A) using conventional AAV2 vectors with overlapping coding sequences, but demonstrated limited success (Lopes *et al*, 2013). Full‐length myosin‐7A was detected, but the level of protein expression was variable, and only a minority of cells showed phenotype correction. More recently, use of dual‐AAV‐TS and dual‐AAV‐Hyb vectors was shown to recombine successfully in HEK cells and in the retinas of mice and pigs (Colella *et al*, 2014; Dyka *et al*, 2014).

As a first attempt in the inner ear, Al‐Moyed *et al* explored the use of dual‐AAV‐TS and dual‐AAV‐Hyb vectors, to deliver cDNA fragments encoding the *Otof* gene into otoferlin‐deficient mice (*Otof* ^−/−^). They showed that single AAV2/6‐GFP vector injection at P6‐7, after the inner ear has already developed, led to successful IHC targeting at an average rate of ~75%. For dual vectors to successfully recombine, both vectors need to transduce the same sensory cell. Thus, with a transduction rate for a single vector of 75%, the predicted dual transduction rate should be ~56%. The investigators reported transduction rates with AAV2/6 dual vectors of 30% and 19% inner hair cells exposed to –TS or –Hyb vectors, respectively. PCR and Western blot analysis revealed presence of full‐length mRNA and protein, respectively, in dual vector‐injected cochleas but not in uninjected *Otof* ^−/−^ cochleas, demonstrating the first successful recombination of the wild‐type form of a large deafness gene in the inner ear. Since otoferlin is involved in synaptic transmission, the investigators counted the number of synapses present between IHCs and SGNs of injected and uninjected *Otof* ^−/−^ mice and found little difference, perhaps suggesting that the time of injection was too late to recover synapse number. However, using an electrophysiological measure of membrane capacitance, they found injected mice had improved synaptic vesicle fusion and presumably neurotransmitter release from IHCs onto SGNs, indicating some recovery of synaptic function.

To investigate whether the synaptic recovery was sufficient to drive transmission of auditory signals along the eighth cranial nerve to the brain, they recorded auditory brainstem responses (ABRs). The ABR is a standard assay that provides an electrical field recording of the compound activity along the ascending auditory pathway. Deaf mice lack ABRs whereas *Otof* ^−/−^ mice injected with dual vectors had sound‐evoked ABRs with average thresholds of 50–60 dB. This is a remarkable level of auditory function given that *Otof* gene replacement requires not just the dual viral transduction but also depends on the efficiency of the recombination event. Prior work suggested ~70% of functional inner hair cells are needed for normal auditory function (Wang *et al*, 1997). In their best performers, Al Moyed *et al* demonstrated dual transduction of up to 51% of IHCs and ABR thresholds as low as 50 dB (WT thresholds range from 20 to 40 dB) (Fig 1), suggesting that with further improvements in efficiency, their dual delivery strategy may approach 70% functional inner hair cells and perhaps normal hearing.

This work is important as the first demonstration of gene therapy for a mouse model of DFNB9, which may affect up to 8% of genetic hearing loss patients. In addition, Al Moyed *et al* provide proof‐of‐principal that dual‐AAV vectors may be suitable for delivery of large transgenes into mammalian IHCs. While only partial recovery of auditory function was observed in treated *Otof* ^−/−^ mice, use of different AAV serotypes, IHC‐specific promoters, enhancers or injection methods may potentially improve outcomes. Further increases in efficiency may allow for recombination of even larger coding sequences, perhaps > 9 kB. For example, Maddalena *et al* (2018) used triple AAV vectors, expanding the capacity up to ~14 kB, to deliver the coding sequence of cadherin‐23 into the retina, albeit with reduced efficiency as expected for triple transduction. The need for efficient inner ear delivery strategies for large coding sequences is abundant, since over 35% of genetic hearing loss is caused by mutations in genes with coding sequences that exceed the capacity of single AAV capsids. Thus, we find it heartwarming to see dual‐AAV delivery of split otoferlin, recombination of the full‐length coding sequence, recovery of synaptic transmission and IHCs and SGNs reunited.

## Conflict of interest

JR Holt is an advisor to several biotech companies focused on inner ear therapies. The authors declare that they have no conflict of interest.
